# Anticholinergic Toxidrome Following Indirect Exposure to Hyoscyamus muticus Through Grasshopper Ingestion: A Case Report

**DOI:** 10.7759/cureus.100166

**Published:** 2025-12-27

**Authors:** Mohammad Halawani

**Affiliations:** 1 Emergency Medicine, Prince Mohammed Bin Abdulaziz Hospital, Madinah, SAU

**Keywords:** anticholinergic syndrome, emergency department, emergency medicine, hyoscyamus muticus, toxicology

## Abstract

Anticholinergic toxidrome is a well-recognized clinical syndrome resulting from the inhibition of muscarinic receptors by substances such as tropane alkaloids, which are found in certain toxic plants like *Hyoscyamus muticus* (Egyptian henbane). While direct plant ingestion is a common cause, indirect exposure through ingestion of animals that have consumed toxic plants is extremely rare.

We report a rare case of a 43-year-old man and his 10-year-old son who presented to the emergency department with symptoms of anticholinergic toxidrome, including blurred vision, dry mouth, nausea, and abdominal discomfort, after ingesting a grasshopper observed feeding on *H. muticus*. The father had no medical history, while the child had a known seizure disorder controlled on medication. Both patients were hemodynamically stable and exhibited classic mild anticholinergic signs. Routine investigations were unremarkable. They were managed conservatively with supportive care, and both recovered fully without the need for antidotal therapy.

This case highlights a rare but important route of exposure to anticholinergic toxins through the food chain via insect ingestion. Clinicians should maintain a high index of suspicion for plant-based toxins in atypical presentations and consider indirect routes of exposure, especially in rural settings. Thorough history-taking remains essential in identifying environmental or dietary sources of toxic exposure.

## Introduction

Anticholinergic toxidrome is a clinical syndrome caused by the inhibition of acetylcholine at muscarinic and, in some cases, nicotinic receptors. This blockade leads to a characteristic group of signs and symptoms, including tachycardia, elevated blood pressure, dry mouth (xerostomia), blurred vision, urinary retention, constipation, and, in severe cases, confusion, delirium, and seizures [1]. The most common sources of anticholinergic toxidrome are medications, such as antihistamines, antipsychotics, and tricyclic antidepressants. However, plants that contain tropane alkaloids, such as *Atropa bella-donna*, *Datura stramonium*, and *Hyoscyamus*, are also well-recognized causes [1].

Insects have been consumed as part of the human diet since ancient times and remain a staple food in many cultures due to their high protein and vitamin content, as well as low fat levels [2]. Although toxicity from direct ingestion of these plants is well documented, cases of indirect exposure through intermediaries are rare. This report describes a rare case of anticholinergic toxidrome in a father and son following the consumption of a grasshopper that was feeding on *Hyoscyamus*.

*Hyoscyamus muticus* and other *Hyoscyamus* species are widely recognized by locals for their toxic potential. These plants are prevalent throughout most regions of Saudi Arabia, making exposure possible nationwide [3].

## Case presentation

A 43-year-old male patient and his 10-year-old son presented to the emergency department (ED) after they ate small portions of grilled grasshoppers. Soon after, they started to experience inappropriate laughter, followed by dry mouth and blurred vision, and abdominal discomfort. All these symptoms began about one hour after eating, as they were hiding at home, and as the symptoms started to worsen, they decided to come to the ED after midnight.

The 43-year-old male patient with no significant medical history presented to the ED with abdominal discomfort, nausea, blurred vision, and dry mouth. Vital signs were blood pressure 135/70 mmHg, heart rate 100 bpm, respiratory rate 16/min, oxygen saturation 98% on room air, temperature 36.8°C, and normal glucose levels. Physical examination revealed mydriasis and dry oral mucous membranes. The remainder of the examination was unremarkable, with no photophobia, urine retention, flushed skin, hallucinations, confusion, or agitation. A 10-year-old boy with a history of seizure disorder managed with valproic acid presented with similar symptoms, including abdominal pain, nausea, blurred vision, and dry mouth. His vital signs were blood pressure 110/70 mmHg, heart rate 110 bpm, respiratory rate 18/min, oxygen saturation 98% on room air, temperature 36.7°C, and normal glucose levels. The physical examination revealed dry oral mucosa, with no signs of urine retention, photophobia, seizure activity, or neurological deficits.

**Figure 1:**
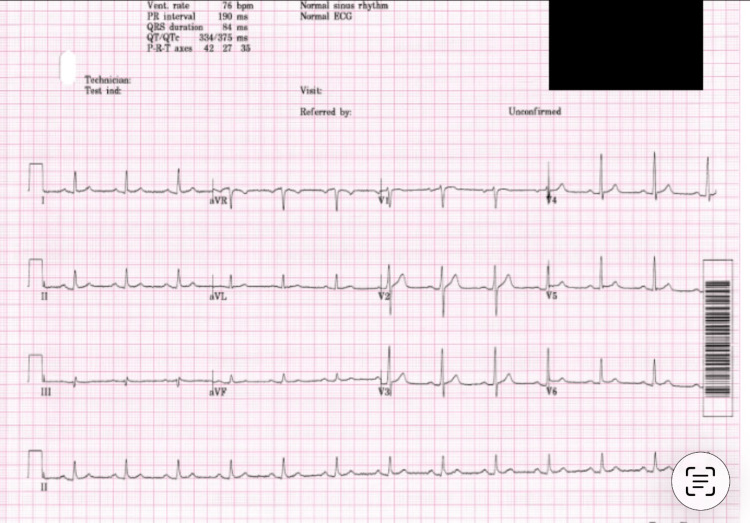
ECG of the 43-year-old male patient

Both patients underwent routine laboratory evaluation, including assessment of electrolytes, renal function, and liver function (Table 1). An electrocardiogram (ECG) (Figure 1) revealed a normal sinus rhythm with no abnormalities. There was no history of taking any medication before the symptoms started, no suspicion of intentional self-harm, so drug levels and other toxicology screens were not performed.

**Table 1:** Laboratory investigations

Test	Patient 1	Patient 2	Normal lab range
Sodium	142 mEq/L	136 mEq/L	135-145 mEq/L
Potassium	4.1 mEq/L	3.7 mEq/L	3.5-5.1 mEq/L
Chloride	103 mEq/L	109 mEq/L	98-111 mEq/L
Calcium	2.4 mmol/L	2.2 mmol/L	2.2-2.6 mmol/L
pH	7.42	7.37	7.35-7.45
Bicarbonate level	21 mEq/L	24 mEq/L	22-26 mEq/L
Creatinine	82 μmol/L	66 μmol/L	55-105 μmol/L
Glucose	6.3 mmol/L	5.6 mmol/L	4.1-5.9 mmol/L

**Figure 2:**
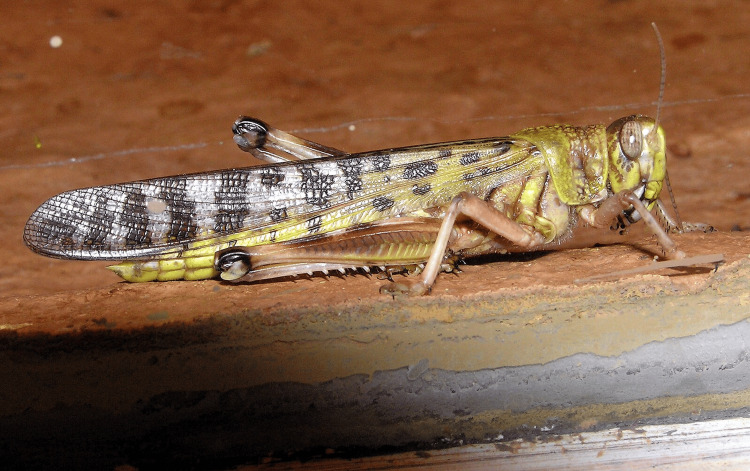
Image of a grasshopper Credit: “Desert locust (*Schistocerca gregaria*)” by Arpingstone, released into the public domain, via Wikimedia Commons.

**Figure 3:**
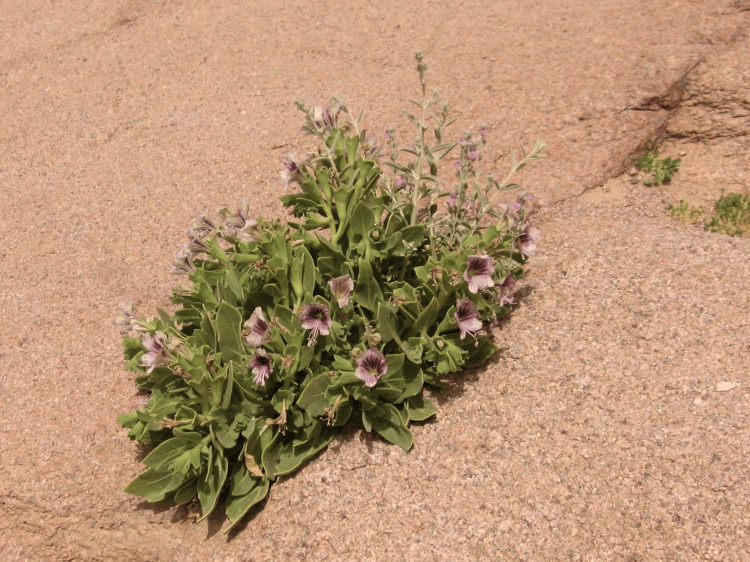
Image of Hyoscyamus muticus L Credit: *Hyoscyamus muticus* by Luca Fornasari, licensed under CC BY-SA 3.0.

Based on the history of ingestion and clinical findings, a diagnosis of anticholinergic toxidrome secondary to consumption of a grasshopper (Figure 2) that had ingested *Hyoscyamus* (Figure 3) was established.

Both patients received supportive care, including intravenous fluids for hydration. Administration of activated charcoal was considered but not done, as around two hours had passed since ingestion. Given the mild clinical presentation and absence of significant neurological or cardiovascular symptoms, administration of pharmacological agents such as physostigmine was deemed unnecessary.

The patients were observed in the ED for four hours. The signs and symptoms gradually improved, with resolution of blurred vision and dry mouth. They were discharged in stable condition. After making sure by taking a deep history about other possibilities that might be the cause of their symptoms, it was clear that their signs and symptoms were all related to their last meal. Both patients made a complete recovery without complications. They were counseled to refrain from consuming unidentified insects or plants and were provided with educational materials regarding the safety of plants and insects.

## Discussion

Anticholinergic toxidrome presents with a constellation of signs and symptoms due to the inhibition of acetylcholine. Classic features include dry mouth, mydriasis, tachycardia, urinary retention, constipation, and confusion. Severe cases may lead to delirium, seizures, or cardiovascular collapse [1].

Plants such as *Hyoscyamus* are rich in tropane alkaloids, which are responsible for anticholinergic effects [4]. Reports of toxicity via indirect ingestion, such as through the consumption of insects that feed on toxic plants, are exceedingly rare. The diagnosis and initial management of anticholinergic toxicity necessitate a systematic toxicological assessment consistent with the workup for any poisoned patient. Starting with the immediate assessment and stabilization of the patient's airway, breathing, and circulation (ABC), this is followed by continuous monitoring of vital signs, including heart rate, respiratory rate, pulse oximetry, blood pressure, and checking the glucose levels [5].

Management is usually supportive, with no antidote in most cases. Benzodiazepines are used in cases of agitation and seizures, and physostigmine may be given if there is no response. Fluids are administered if the patient is hypotensive or has rhabdomyolysis. Some patients may require external cooling to manage hyperthermia. Activated charcoal may be used if ingestion occurred within one hour of presentation [6]. Because of the mild signs and symptoms in our patients and the fact that they presented to the ED almost two hours after ingestion, only fluids were given.

In this case, the grasshopper that had consumed *Hyoscyamus* likely retained tropane alkaloids in its tissues, resulting in mild anticholinergic toxicity in the patients. In this case, the patients consumed only a small amount of the insect. It is uncertain whether ingestion of a larger quantity would have resulted in more severe toxicity.

This case highlights the importance of considering environmental factors, including indirect exposures, in evaluating toxidrome presentations. Clinicians should be vigilant for atypical routes of toxin exposure, particularly in rural or wilderness settings where contact with plants and insects is common.

## Conclusions

This case report describes a rare instance of mild anticholinergic toxidrome following ingestion of a grasshopper that had consumed *Hyoscyamus* plants. Both patients experienced mild poisoning and recovered fully with supportive care. The case highlights the risk of indirect plant toxin exposure through the food chain and emphasizes the importance of thorough history taking in toxicology assessments. Implementing community education on the identification and dangers of toxic plants, as well as agricultural monitoring programs, could significantly reduce the incidence of such cases. Increasing awareness and preventive strategies in rural settings may serve as important public health measures.
